# Hypoglycemic Effects of *Lycium barbarum* Polysaccharide in Type 2 Diabetes Mellitus Mice *via* Modulating Gut Microbiota

**DOI:** 10.3389/fnut.2022.916271

**Published:** 2022-06-30

**Authors:** Qingyu Ma, Ruohan Zhai, Xiaoqing Xie, Tao Chen, Ziqi Zhang, Huicui Liu, Chenxi Nie, Xiaojin Yuan, Aobai Tu, Baoming Tian, Min Zhang, Zhifei Chen, Juxiu Li

**Affiliations:** College of Food Science and Engineering, Northwest A&F University, Yangling, China

**Keywords:** *Lycium barbarum* polysaccharide, type 2 diabetes mellieus, gut microbiota, hypoglycemia, hypolipidemia

## Abstract

This study aims to explore the molecular mechanisms of *Lycium barbarum* polysaccharide (LBP) in alleviating type 2 diabetes through intestinal flora modulation. A high-fat diet (HFD) combined with streptozotocin (STZ) was applied to create a diabetic model. The results indicated that LBP effectively alleviated the symptoms of hyperglycemia, hyperlipidemia, and insulin resistance in diabetic mice. A high dosage of LBP exerted better hypoglycemic effects than low and medium dosages. In diabetic mice, LBP significantly boosted the activities of CAT, SOD, and GSH-Px and reduced inflammation. The analysis of 16S rDNA disclosed that LBP notably improved the composition of intestinal flora, increasing the relative abundance of *Bacteroides*, *Ruminococcaceae_UCG-014*, *Intestinimonas*, *Mucispirillum*, *Ruminococcaceae_UCG-009* and decreasing the relative abundance of *Allobaculum*, *Dubosiella*, *Romboutsia*. LBP significantly improved the production of short-chain fatty acids (SCFAs) in diabetic mice, which corresponded to the increase in the beneficial genus. According to Spearman’s correlation analysis, *Cetobacterium*, *Streptococcus*, *Ralstonia*. *Cetobacterium*, *Ruminiclostridium*, and *Bifidobacterium* correlated positively with insulin, whereas *Cetobacterium*, *Millionella*, *Clostridium_sensu_stricto_1*, *Streptococcus*, and *Ruminococcaceae_UCG_009* correlated negatively with HOMA-IR, HDL-C, ALT, AST, TC, and lipopolysaccharide (LPS). These findings suggested that the mentioned genus may be beneficial to diabetic mice’s hypoglycemia and hypolipidemia. The up-regulation of peptide YY (PYY), glucagon-like peptide-1 (GLP-1), and insulin were remarkably reversed by LBP in diabetic mice. The real-time PCR (RT-PCR) analysis illustrated that LBP distinctly regulated the glucose metabolism of diabetic mice by activating the IRS/PI3K/Akt signal pathway. These results indicated that LBP effectively alleviated the hyperglycemia and hyperlipidemia of diabetic mice by modulating intestinal flora.

## Graphical Abstract

Effects of *Lycium barbarum* polysaccharide on glucose homeostasis in diabetic mice. 
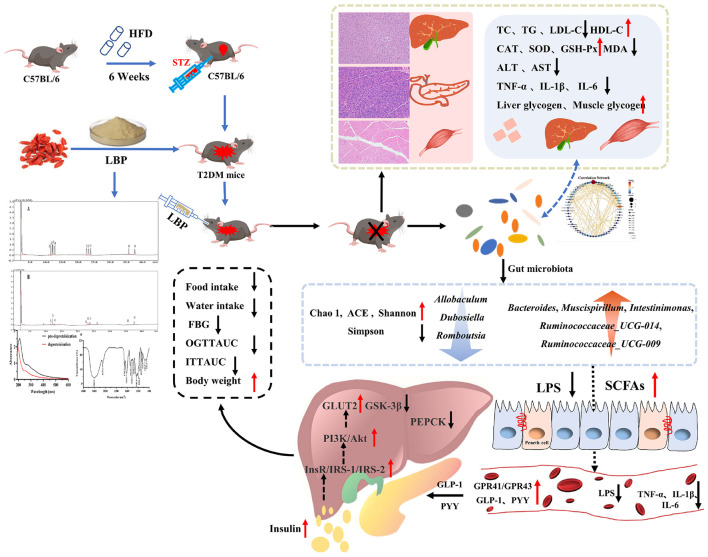

## Introduction

The global burden of diabetes mellitus has risen rapidly in recent decades, however, many individuals go undiagnosed (1). According to the latest research by the International Diabetes Federation (IDF, 10th edition), the global diabetes prevalence is expected to be 10.5% (537 million) in 2021, rising to 12.2% (783 million) in 2045 (2). Diabetes mellitus is a chronic metabolic condition that has been linked to several factors, including age, genetic predisposition, lifestyle, dietary patterns, as well as physical and mental health (3). Type 1 diabetes mellitus (T1DM), type 2 diabetes mellitus (T2DM), and gestational diabetes mellitus (GDM) are the three basic kinds of diabetes (2). T2DM is defined as a relative insulin shortage induced by pancreatic β-cell dysfunction and failure, which gradually leads to insulin resistance in the tissues, liver, and skeletal muscle over time (4). T2DM accounts for nearly 90% of the total and the global burden of diabetes is expected to reach 1,054 billion USD by 2045, accompanied by increased health expenditures for public care of this disease (2). Long-time high blood glucose exposure elevated the development of reactive oxygen species (ROS), which could be harmful to the host’s health (5). Several factors, including food, lifestyle changes, medication, and bariatric surgery, can prevent or delay the onset and progression of T2DM. Metformin, insulin secretagogues, glycosidase inhibitors, glucagon-like peptide-1 (GLP-1), analogs, DPP-4 inhibitors, SGLT-2 inhibitors, as well as insulin and its agonists are used to treat diabetes (6). Although therapeutic medicines are efficient at managing blood glucose levels, they can also cause a variety of side effects, including blood glucose fluctuations, hypoglycemia, gastrointestinal reactions, and liver, pancreatic, and kidney injuries (7). As a result, researchers must devise a new strategy to combat T2DM.

The growing evidence has implicated that host health is inextricably linked to intestinal flora (8). Some studies have linked the altered intestinal flora composition to the onset of obesity, diabetes, peripheral tissue inflammation, and gastrointestinal disorders (9, 10). The disorder and dysfunction of intestinal flora may lead to an increase of opportunistic pathogens, such as *Betaproteobacteria* and *Clostridium bolteae*, which may be able to damage the host (9). The decline of *Faecalibacterium prausnitzii* could reduce the production of SCFAs, which should be accompanied by an increase in destructive inflammatory cytokines (10). Intestinal flora dysbiosis can cause tissue and organ dysfunction, interfere with endogenous metabolites in the host, and cause other harm to the organism (11). Polysaccharides have been shown to play an important role in the prevention of the onset and progression of T2DM by modulation of intestinal flora (12–14). Polysaccharides isolated from *Vigna angularis* and mulberry leaf alleviated diabetes in diabetic rats by activating the signaling pathway of insulin/PI3K/AKT or blocking islet cell apoptosis (15). Furthermore, *Ganoderma lucidum* polysaccharide reversed gut dysbiosis and decreased the Firmicutes–Bacteroidetes ratio (16). Another study found that by altering the composition of intestinal flora, *G. lucidum* polysaccharide reduced inflammation, and delayed the onset and progress of diabetes (17). Polysaccharides isolated from *Holothuria leucospilota* significantly increased the proportion of SCFAs-producing bacteria in diabetic mice, including *Lactobacillus* and *Bifidobacterium* (14). These studies revealed that polysaccharides may be useful in regulating the composition of intestinal flora and selectively increased the proportion of beneficial bacteria.

Type 2 diabetes mellitus is a chronic metabolic disease characterized by uncontrolled insulin secretion, hyperglycemia, and hyperlipidemia (1). Many recent studies have demonstrated that *Lycium barbarum* polysaccharide (LBP) could help with T2DM in a variety of ways (18, 19). Some reports have shown that LBP can influence the composition and structure of gut microbiota (18–21). *Lycium barbarum* is a well-known traditional Chinese herb and is a popular food in China and other Asian countries (22). The concentrated juice or extracts of this fruit are added to beverages to improve liver function and reduce oxidative stress damage (21). LBP is regarded as one of the most important active components with numerous biological activities, including antioxidant, anti-tumor, anti-inflammatory, anti-hyperglycemic, and anti-hyperlipidemic properties (21, 22). LBP’s biological activities are primarily determined by its chemical structure, chain conformations, and molecular weight (MW) (23). LBP has a MW range of 10–2,300 kDa (24). Although different extraction methods cause variations in the composition of LBP, its monosaccharide mainly is primarily composed of xylose (Xyl), glucose (Glc), rhamnose (Rha), mannose (Man), galactose (Gal), arabinose (Ara), fructose (Fru), fucose (Fuc), ribose (Rib), galacturonic acid (GalA), and glucuronic acid (GlcA) (24). Furthermore, the biological activities of LBP are also closely related to its chemical structures. A reported review summarized that the backbones of LBP are mainly composed of (1 → 3)-β-Gal*p*, (1 → 4)-β-Gal*p*, (1 → 6)-β-Gal*p*, (1 → 6)-α-glucans, and (1 → 4)-α polygalacturonans with various branches and terminals (24). Even though it has been well documented that LBP can alleviate diabetic symptoms by modulating intestinal flora. However, the potential molecular mechanism of LBP in diabetes alleviation from the perspective of the gut microbiota has not been fully elucidated. As a result, the goal of this study is to gain a better understanding of the molecular mechanism of LBP by modulating intestinal flora in the treatment of T2DM. The LBP’s hypoglycemic and hypolipidemic effects, as well as its modulation of the gut microbiota, were investigated. This study estimated the mRNA expression of critical genes involved in glucose metabolism. Furthermore, we performed a Spearman correlation between gut microbiota and biochemical parameters. These findings may help to elucidate the potential mechanism of LBP in the treatment of diabetes and its complications.

## Materials and Methods

### Materials and Reagents

The dried fruits of *L. barbarum* were provided by the National Wolfberry Engineering Research Center of Ningxia Academy of Agricultural and Forestry Sciences (Yinchuan, China). 3-Methyl-1-phenyl-2-pyrazoline-5-one (PMP), monosaccharide standards, and streptozotocin (STZ) were obtained from Sigma Chemical Co., Ltd. (St. Louis, MO, United States). Standards of SCFAs were obtained from Shanghai Yuanye Biotechnology Co., Ltd. (Shanghai, China). A commercial company supplied the mouse feeds (Trophic Animal Feed High-Tech Co., Ltd., Nantong, China). All the other chemicals and solvents used in this study were analytical grade and obtained from Sinopharm Chemical Reagent Co., Ltd. (Shanghai, China).

### Preparation of *Lycium barbarum* Polysaccharide

*Lycium barbarum* polysaccharide was prepared by referring to the works of literature with appropriate modifications (25–27). Briefly, the dried powder of *L. barbarum* was treated with ethanol solution (v/v, 80%) for 2 h to remove small molecules, e.g., monosaccharides, oligosaccharides, pigments, and polyphenols, as well as alcohol-soluble fats, before drying the residues at 60°C overnight. The extraction procedure was as follows: the dried residues were mixed with distilled water (w/v, 1:20) and ultrasonicated (60°C, 180 W) for 15 min before being extracted at 90°C for 105 min. The extraction procedure described above was repeated two times and combined. The extracts were concentrated under reduced pressure at 50°C using a rotary evaporator. The concentrated solution was mixed with absolute ethanol (v/v, 1:4) overnight (4°C). Centrifugation was used to obtain the precipitate (3,500 rpm, 10 min). Repeat the concentrated operation three times after resolving in deionized water. The reported method was used to remove proteins (28). Finally, after removing the small molecule with a dialysis membrane (3.5 kDa), the mixture solution was collected, concentrated, and lyophilized to obtain LBP.

### Characterization of *Lycium barbarum* Polysaccharide

The total carbohydrates, protein, and uronic acid contents were determined using previously published methods (29–31). A UV-3100 spectrophotometer was used to scan the UV spectrum of LBP (Mapada Inc., Shanghai, China). LBP samples were formulated with ultrapure water (1 mg/ml) and scanned at the wavelengths ranging from 190 to 610 nm, with ultrapure water served as a blank control (32). The Fourier-transformed infrared (FTIR) spectra were obtained using the Bruker Vetex70 FT-IR instrument (Vetex70, Bruker Co., Germany) within the frequency range of 4,000–400 cm^–1^ (33). The MW of LBP was determined using Infinity Liquid Chromatography (Agilent Technologies Inc., Wilmington, DE, United States) equipped with the TSKgel G5000PWXL column (7.5 mm × 30 cm; TOSOH Corporation, Japan) and the Agilent 1260 refractive index detector (34). In brief, the flow phase was ultrapure water (0.5 ml/min), LBP was formulated at 2.0 mg/ml and a 10 μl solution was injected to be analyzed; the column temperature was kept at 30°C. The calibration equation was lg MW = −0.3259t + 10.949 (*R*^2^ = 0.9957) based on the retention of standards dextran. The monosaccharide composition of LBP was analyzed by GC–MS, and the procedure was carried out using chromatography (Shimadzu 2014C, Japan) equipped with the DB-17 column (30 m × 0.25 mm × 0.25 μm) (35). The monosaccharide standard mixture was consistently treated with LBP.

### Animal Experiments

**FIGURE 1:**
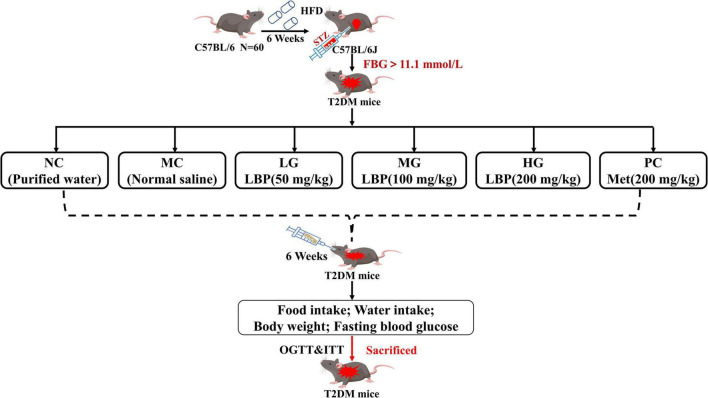
Animal experimental protocol and design. All the mice were divided into a normal group (administrated with normal saline) and diabetic groups (injected with multiple low dosages of STZ at the beginning of the seventh week, 40 mg/kg/day × 3). The diabetic mice were divided randomly into five groups: model control group (MC), low dosage group (LG), medium dosage group (MG), high dosage group (HG), and positive control group (PC).

The specific pathogen-free C57BL/6 mice (male, 6-week-old, SCXK-2018-001) were provided by Xi’an Jiaotong University (Xi’an, China). All mice were housed in accredited animal facilities in an environment with a 12-h reverse light–dark cycle (temperature 22 ± 2°C, humidity 55 ± 5%). Figure 1 depicts the experiment after 1 week of adaptation, which was carried out using previous methods with appropriate modifications (32). Mice fed a high-fat diet (HFD, TP 23300, 60% kcal from fat) were prepared for insulin resistance in mice, while mice in the normal control group (NC) were fed with normal feed (LAD 3001G, 16.7% kcal from fat). All mice were fed with HFD for 6 weeks and then starved for 6 h before receiving an intraperitoneal injection of fresh-prepared STZ buffer (pH 4.2–4.5, 0.1 mol/L in sodium citrate solution, 40 mg/kg) within 30 min. Mice in the normal control group received the same volume of sodium citrate solution. After 72 h, the level of fasting blood glucose (FBG) was measured using a glucose analyzer, and FBG above 11.1 mmol/L was considered in diabetic mice (36). Food and water intake, body weight (BW), and FBG levels in mice were all recorded. All mice were anesthetized and executed, and the main organs were weighed after the removal of surface blood. All experimental procedures were approved by the Animal Ethics Committee of Xi’an Jiaotong University.

### Oral Glucose Tolerance Test and Insulin Tolerance Test

The oral glucose tolerance test (OGTT) and insulin tolerance test (ITT) were performed in the final week of the animal experiment (37). OGTT loaded glucose (2 g/kg BW) by gavage after 12-h fasting. FBG levels in blood from the tail vein were measured before and after glucose administration at 15, 30, 60, 90, and 120 min. The mice were fasting for 4 h, ITT was conducted by injecting intraperitoneally (0.5 IU/kg BW) and then measuring FBG in blood from the tail vein at the same point in time as OGTT. As described previously (38), the homeostasis model assessment for insulin resistance (HOMA-IR) and homeostasis model assessment for beta cell (HOMA-β) was calculated as (FBG × INS)/22.5, and 20 × INS/(FBG − 3.5), respectively.

### Biochemical Parameter Analysis

Levels of glycated serum protein (GSP), glycogen (liver and muscle), and other biochemical parameters were determined with commercial kits purchased from the Nanjing Jiancheng Bioengineering Institute (Nanjing, China). ELISA kits were used to measure hemoglobin A1c (HbA1c), LPS, interleukin-6 (IL-6), interleukin-1β (IL-1β), GLP-1, peptide YY (PYY), tumor necrosis factor-α (TNF-α), and insulin (INS) (Colorful Gene, Wuhan, China).

### Histopathological Analysis of Liver, Pancreas, and Skeleton Muscle

Histopathological examinations of the liver, pancreas, and skeletal muscle were performed by previously reported methods (32). A small section of the tissues was fixed in 10% formalin, dehydrated in alcohol, embedded in paraffin, and stained with hematoxylin–eosin (H&E).

### Quantification of Short-Chain Fatty Acids in Fecal Samples

Gas chromatography was used to determine SCFAs in feces samples based on previously reported methods with appropriate modifications (39). In brief, approximately 200 mg of feces were dissolved with ultrapure water (w/v, 1:5) in a 5 ml centrifuge tube, and dissolved material from the vortexes was mixed thoroughly. Afterward, the mixture was centrifuged (12,000 rpm, 10 min), 0.75 ml supernatant fluid was mixed with 0.15 ml H_2_SO_4_ (v/v), and then mixed with 1.6 ml of diethyl ether, followed by incubating in shaking table (160 rpm, 30 min), while simultaneously mixing 10 times. The mixed solution was then centrifuged for 10 min at 4°C and 12,000 rpm to collect the organic phase in the upper layer. The organic phase was concentrated with liquid nitrogen from 1.5 to 0.3 ml and then passed through a 0.25 μm organic filter for further analysis as described below: the initial temperature was 50°C (1 min) and raised to 120°C (15°C/min), increased to 170°C (5°C/min), raised to 220°C (15°C/min, maintained 5 min), injection volume 2.0 μl. The temperatures of the injector and detector were 250 and 270°C, respectively.

### 16S rDNA Sequencing and Bioinformatics Analysis

Fresh fecal pellets from eight mice per group were collected for microbial analysis of intestinal flora (40). According to the manufacturer’s instructions, bacterial genomic DNA extracted from feces was obtained by a QIAamp DNA stool Mini Kit from Qiagen (Hilden, Germany). The V3 and V4 regions of 16S rDNA were amplified by 338F (5′-ACTCCTACGGGCAGCAG-3′) and 806R (5′-GGACTACHVGGGTWTCTAAT-3′) universal primers with dual-index barcodes to tag each sample. The concentrations of PCR products were measured using a QuantiFluor-St Fluorometer (Promega, United States). The fecal samples of PCR products in equimolar concentrations were examined on the Illumina MiSeq platform following the manufacturer’s manual by Shanghai Biotree Biomedical Technology Co., Ltd. (Shanghai, China).

### Quantitative Real-Time PCR Analysis

Total RNA was extracted from colon and liver tissues using the TRIzol reagents (Jingcai Bio., Xi’an, China), and reverse transcription reagents were used to synthesize cDNA (Tiangen, Beijing, China). Real-time PCR (RT-PCR) was used to measure gene expression on a CFX96 RT-PCR detection system (Bio-Rad, Hercules, CA, United States) using the SYBR Green master mix (Tiangen, Beijing, China). The 2^–ΔΔ^*^CT^* method was used to normalize gene expression to that of the housekeeping gene (β-actin). Supplementary Table 1 shows the primer sequences used in this study.

### Statistical Analysis

Statistical analysis of data was analyzed by SPSS V22.0 software (IMB, Chicago, IL, United States). The differences were conducted using a one-way analysis of variance (ANOVA) test followed by Duncan’s test. Results were presented as mean ± SD. Values with *p* < 0.05 were considered to be statistically significant.

## Results and Analysis

### Characterization of *Lycium barbarum* Polysaccharide

**FIGURE 2:**
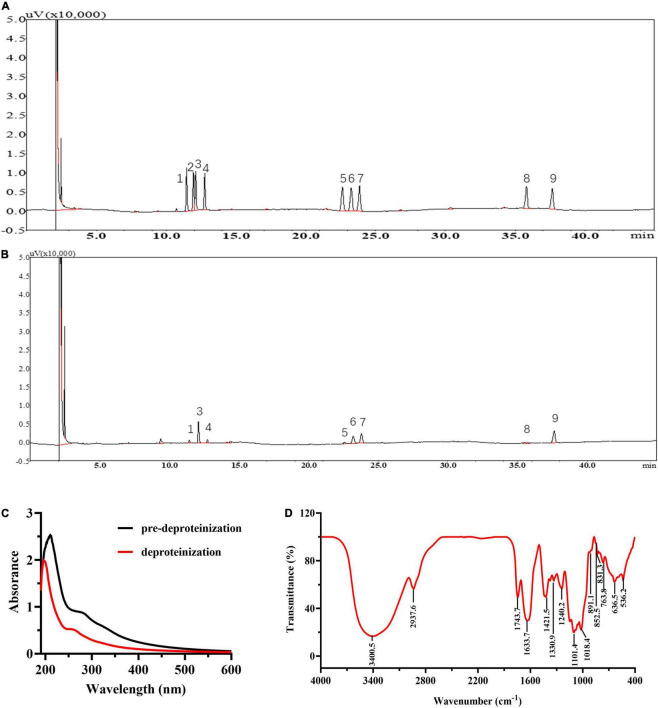
Chemical information of LBP. GC chromatogram of the monosaccharide standards **(A)**, and LBP **(B)**. The peaks in monosaccharide standards and LBP represent (1) rhamnose, (2) fructose, (3) arabinose, (4) xylose, (5) mannose, (6) glucose, (7) galactose, (8) glucuronic acid, (9) galacturonic acid; the **(C)** UV spectra of LBP, **(D)** FTIR spectra of LBP.

The yield of LBP is 2.76 ± 0.33%. LBP mainly consisted of carbohydrates, uronic acid, and protein of 62.27 ± 2.37, 25.03 ± 1.27, and 2.92 ± 0.17%. Figures 2A,B show the GC chromatograms of the monosaccharide standards and LBP, respectively. The GC chromatogram in Figure 2B revealed that LBP was made up of eight monosaccharides, with a molar ratio of 0.23: 1.90: 0.26: 0.20: 1.0: 1.26: 0.44: 1.49 for Rha, Ara, Xyl, Man, Glc, Gal, GlcA, GalA residues. The retention time (18.283 min) was used to compute the MW of LBP, which was found to be 98.0 kDa. In Figure 2C, UV spectroscopy examination of LBP revealed no significant absorption at 260–280 nm, indicating that the LBP does not include protein or nucleic acid (32). Figure 2D shows the results of FT-IR spectroscopy used to characterize LBP. Polysaccharide absorption peaks were found at 800–1,200, 1,450–1,800, 2,900–3,000, and 3,200–3,600 cm^–1^ in the spectra. The hydroxyl group (O-H) had a characteristic significant broad stretch peak at 3,400.5 cm^–1^, while the C-H stretching vibration had a band at 2,937.6 cm^–1^ (41). The broadband at 1633.7 cm^–1^ was created by bound water (18). The absorbance of a carboxylic group (COO-) is responsible for a strong band of 1,743.7 cm^–1^ and a weak band of 1,421.5 cm^–1^, indicating that LBP included uronic acid (35). The C = O stretching vibration is responsible for the frequency band at 1330.9 cm^–1^ (42). The fingerprint of molecules is a wavelength range between 950 and 1,200 cm^–1^ that is used to determine the position and intensity of distinctive bands in polysaccharides (43). The presence of C-O bonds and furanose rings in the monosaccharide blocks of LBP is attributed to the intense stretching peaks at 1,101.4 and 1,018.4 cm^–1^. The presence of α-glycosidic, β-glycosidic linkages, and pyranose rings in the monosaccharide blocks of LBP was confirmed by the band at 831.3, 852.5, and 891.1 cm^–1^, respectively. The α-glycosidic bond is prevalent in LBP, as evidenced by the moderate absorbance at 831.3 and 852.5 cm^–1^, and the weak absorbance at 891.1 cm^–1^ (18).

### Effects of *Lycium barbarum* Polysaccharide on Body Weight, Food Intake, and Water Intake in Mice

**FIGURE 3:**
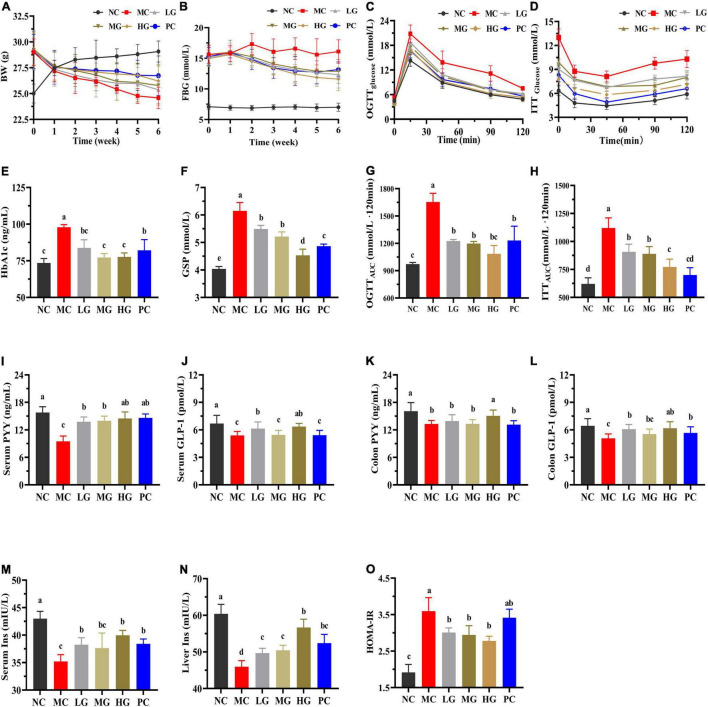
Effects of LBP on biochemical parameters. **(A)** BW; **(B)** FBG; **(C)** OGTT; **(D)** ITT; **(E)** HbA1c; **(F)** GSP; **(G)** OGTTAUC; **(H)** ITTAUC; **(I)** serum PYY; **(J)** serum GLP-1; **(K)** colon PYY; **(L)** colon GLP-1; **(M)** serum INS; **(N)** liver INS; and **(O)** HOMA-IR. Different letters denote significant differences at *p* < 0.05.

Figure 3A and Supplementary Table 2 show that mice in the MC group consumed more food and water intake than that in the NC group (*p* < 0.05). In diabetic mice, LBP reversed BW loss and reduced food and water intake (*p* < 0.05). In comparison with the NC group, the MC group’s total food and water intake increased by 23.44 and 36.30%, respectively. Total food intake reduced by 5.11, 9.22, 14.18, and 11.84% in the LG, MG, HG, and PC groups, respectively, compared with the MC group, while total water intake declined by 17.42, 20.26, 22.60, and 13.55%. These findings showed that LBP reduced BW loss, and food and water intake in diabetic mice.

### Effects of *Lycium barbarum* Polysaccharide on Fasting Blood Glucose, Oral Glucose Tolerance Test, Insulin Tolerance Test, HbA1c, Glycated Serum Protein, Glucagon-Like Peptide-1, Peptide YY, Insulin, and HOMA-IR in Mice

Figure 3B shows that FBG in the MC group increased by 149.57% when compared with the NC group. FBG levels in the LG, MG, HG, and PC groups decreased by 24.25, 25.52, 33.02, and 26.50%, respectively, as compared with the MC group. In Figures 3C,D, LBP notably reduced the levels of OGTTAUC, ITTAUC, and HOMA-IR Figure 3O in comparison with the MC group (*p* < 0.05). As shown in Figure 3C, after loading glucose, the blood glucose levels reached a peak in each group within 30 min. Furthermore, at each time point, blood glucose in the MC group was higher than that in the other groups in (Figures 3G,H). LBP significantly reduced OGTTAUC and ITTAUC levels in the LG, MG, and HG groups compared with the NC group (*p* < 0.05), with a decrease of 25.98, 27.67, and 34.50% (OGTTAUC), 18.99, 20.62 and 31.04% (ITTAUC), respectively. HbA1c (Figure 3E), GSP (Figure 3F), and HOMA-IR (Figure 3O) levels were significantly higher in the MC group than those in the NC group, while GLP-1 (Figures 3J,L), PYY (Figures 3I,K), and insulin (Figures 3M,N) levels were significantly lower in the MC group than those in the NC group (*p* < 0.05). As compared with the MC group, LBP reduced HbA1c, GSP, and HOMA-IR levels, improved GLP-1 and PYY levels (serum and colon), and decreased insulin (serum and liver) (*p* < 0.05). These results disclosed that LBP improved insulin sensitivity, alleviated insulin resistance, and improved blood glucose metabolism in diabetic mice.

### Effects of *Lycium barbarum* Polysaccharide on Blood Lipids, Oxidative Stress, and Cytokines in Mice

Table 1 shows that the NC group’s TC, TG, and LDL-C levels were significantly lower than those in the MC group (*p* < 0.05), while TBA and HDL-C levels notably increased (*p* < 0.05). LBP significantly decreased TC, TG, and LDL-C levels (*p* < 0.05), and improved TBA and HDL-C levels in diabetic mice compared to the MC group (*p* < 0.05), indicating that LBP alleviated dyslipidemia. Furthermore, the MC group’s increment of AST and ALT was significantly higher than that in the NC group (*p* < 0.05). When compared to the MC group, LBP remarkably reduced the levels of ALT and AST (*p* < 0.05). These results demonstrated that LBP effectively reversed the abnormal lipid metabolism in diabetic mice.

**TABLE 1:** Effects of LBP on levels of biochemical parameters in serum and liver.

		Group					
		
	Items	NC	MC	LG	MG	HG	PC
Serum	TC (mmol/L)	2.57 ± 0.14 c	3.83 ± 0.07 a	2.89 ± 0.14 bc	2.81 ± 0.27 b	2.68 ± 0.12 bc	2.74 ± 0.11 c
	TG (mmol/L)	0.63 ± 0.07 c	1.05 ± 0.06 a	6.80 ± 0.13 bc	0.88 ± 0.06 b	0.80 ± 0.10 bc	0.73 ± 0.05 c
	LDL-C (mmol/L)	2.32 ± 0.20 d	3.57 ± 0.18 a	2.86 ± 0.14 bc	2.99 ± 0.07 b	2.48 ± 0.24 cd	2.64 ± 0.14 c
	HDL-C (mmol/L)	3.72 ± 0.19 a	2.33 ± 0.13 d	2.96 ± 0.05 c	2.88 ± 0.08 c	3.42 ± 0.15 b	2.45 ± 0.21 d
	ALT (U/L)	7.04 ± 0.61 c	14.90 ± 1.59 a	10.80 ± 1.48 b	10.64 ± 1.40 b	10.32 ± 1.11 b	8.95 ± 1.50 bc
	AST (U/L)	8.40 ± 0.81 d	15.15 ± 1.11 a	12.56 ± 0.97 b	12.11 ± 0.95 b	10.28 ± 0.41 c	13.10 ± 0.67 b
	TBA (μmol/L)	3.35 ± 0.36 a	2.16 ± 0.05 c	2.75 ± 0.19 b	2.78 ± 0.15 b	2.72 ± 0.13 b	2.76 ± 0.10 b
Liver	TC (mmol/gprot)	0.11 ± 0.01 e	0.29 ± 0.02 a	0.19 ± 0.01 c	0.18 ± 0.02 c	0.15 ± 0.01 d	0.24 ± 0.03 b
	TG (mmol/gprot)	0.056 ± 0.004 b	0.128 ± 0.011 a	0.08 ± 0.009 b	0.065 ± 0.012 b	0.060 ± 0.008 b	0.071 ± 0.014 b
	LDL-C (mmol/gprot)	0.074 ± 0.006 b	0.094 ± 0.004 a	0.079 ± 0.003 b	0.079 ± 0.004 b	0.071 ± 0.009 b	0.087 ± 0.003 ab
	HDL-C (mmol/gprot)	0.050 ± 0.001 a	0.035 ± 0.003 c	0.044 ± 0.002 b	0.044 ± 0.003 b	0.043 ± 0.002 b	0.050 ± 0.003 ab
	ALT (U/gprot)	4.77 ± 0.92 d	10.76 ± 1.20 a	8.05 ± 0.74 b	6.46 ± 0.70 c	7.12 ± 0.47 bc	7.79 ± 0.66 bc
	AST (U/gprot)	4.47 ± 0.90 b	8.31 ± 1.34 a	6.02 ± 0.86 b	5.43 ± 0.66 b	5.06 ± 0.53 b	6.66 ± 1.12 ab
	TBA (μmol/L)	16.13 ± 0.65 a	12.47 ± 0.54 b	13.46 ± 0.72 b	15.06 ± 0.69 a	15.61 ± 1.07 a	13.67 ± 0.85 b

*Values are presented as the mean ± SD. Different letters denote significant differences at p < 0.05.*

In Table 2, the levels of CAT, SOD, GSH, GSH-Px, and TAOC (serum and liver) in the MC group were significantly lower than that in the NC group (*p* < 0.05), while the level of MDA was higher than that in the NC group (*p* < 0.05). LBP exhibited an ascendency in promoting the levels of CAT, SOD, GSH-Px, and TAOC, while decreasing MDA in the liver as compared with the MC group (*p* < 0.05).

**TABLE 2:** Effects of LBP on levels of oxidative stress parameters in mice.

		Group					
		
	Items	NC	MC	LG	MG	HG	PC
Liver	CAT (U/mgprot)	31.08 ± 1.80 a	20.12 ± 1.89 c	24.10 ± 1.49 b	25.32 ± 1.72 b	29.92 ± 2.71 a	25.20 ± 1.62 b
	SOD (U/mgprot)	196.20 ± 13.82 a	136.15 ± 4.67 c	174.33 ± 8.86 b	166.42 ± 9.29 b	173.14 ± 13.64 b	177.72 ± 10.52 b
	GSH (μmol/gprot)	4.47 ± 0.27 a	2.99 ± 0.23 c	3.63 ± 0.19 bc	3.72 ± 0.22 bc	3.96 ± 0.24 b	3.32 ± 0.31 c
	GSH-Px (U/gprot)	285.28 ± 7.33 a	203.61 ± 11.81 d	234.55 ± 10.88 c	240.61 ± 9.17 bc	252.26 ± 11.42 b	244.60 ± 9.37 bc
	MDA (nmol/mgprot)	1.28 ± 0.03 a	1.78 ± 0.08 a	1.61 ± 0.17 b	1.46 ± 0.07 bc	1.41 ± 0.05 c	1.46 ± 0.08 bc
	TAOC (mmol/gprot)	0.084 ± 0.012 a	0.050 ± 0.005 c	0.058 ± 0.004 c	0.059 ± 0.005 c	0.072 ± 0.004 b	0.068 ± 0.002 bc
Serum	TAOC (mM Trolox)	0.68 ± 0.03 a	0.51 ± 0.03 b	0.65 ± 0.01 ab	0.67 ± 0.05 a	0.67 ± 0.01 a	0.61 ± 0.02 b

*Values are presented as the mean ± SD. Different letters denote significant differences at p < 0.05.*

**FIGURE 4:**
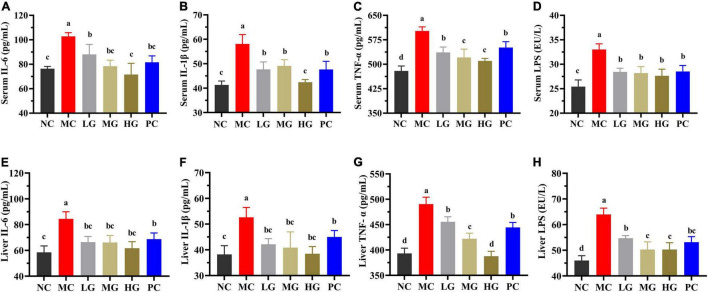
Effects of LBP on **(A)** serum IL-6; **(B)** serum IL-1β; **(C)** serum TNF-α; **(D)** serum LPS; **(E)** liver IL-6; **(F)** liver IL-1β; **(G)** liver TNF-α; and **(H)** liver LPS. Different letters denote significant differences at *p* < 0.05.

In Figure 4, levels of IL-6, IL-1β, TNF-α, and LPS in the MC group were significantly higher than those in the NC group (*p* < 0.05). In comparison to the MC group, LBP significantly reduced the levels of IL-6, IL-1β, TNF-α, and LPS (serum and liver) in the LG, MG, and HG groups (*p* < 0.05), demonstrating that LBP effectively relieved the inflammation of diabetic mice. According to the results, LBP markedly mitigated dyslipidemia, oxidative stress, and inflammation in diabetic mice.

### Histopathological Observations and Glycogen Measurements

**FIGURE 5:**
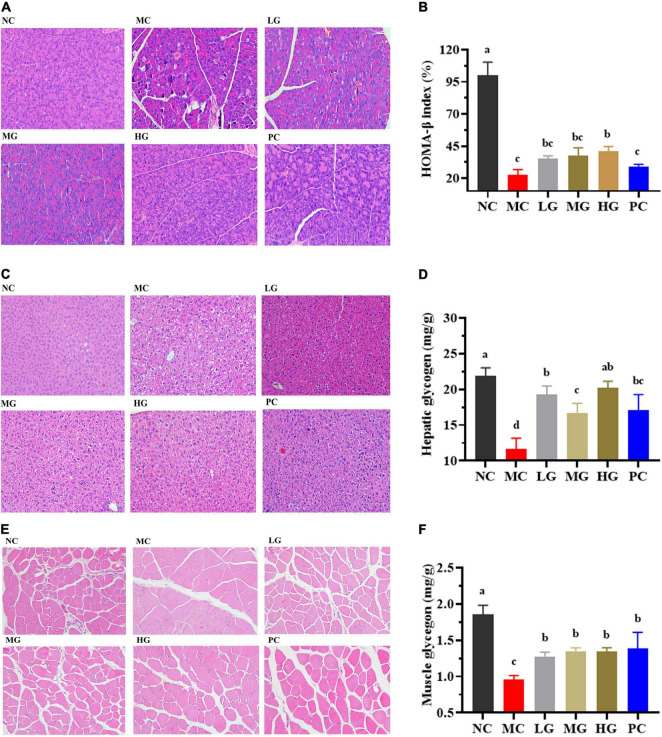
Effects of LBP on pancreas, liver, skeletal muscle, hepatic glycogen, and muscle glycogen. **(A)** H&E staining of pancreas; **(B)** HOMA-β; **(C)** H&E staining of liver; **(D)** hepatic glycogen; **(E)** H&E staining of muscle; and **(F)** muscle glycogen. Different letters denote significant differences at *p* < 0.05.

H&E stating images of the panceras, liver, and skeletal muscle sections are shown in Figure 5. In Figure 5A, the pancreas staining in the NC group exhibited a uniform arrangement of structure and the pancreatic islets contained several pancreatic β-cells, whereas the pancreatic islet in the MC group displayed significant damage compromising and deforming its shape accompanied by a reduction in pancreatic β-cells. LBP partially repaired the damaged state and improved the number of pancreatic β-cells. In Figure 5B, the level of HOMA-β in the NC group was significantly lower than that in the MC group (*p* < 0.05). After treatment of LBP, the HOMA-β level in the HG group was significantly decreased as compared with the MC group (*p* < 0.05). In Figure 5C, the liver cells were normal and neatly arranged without clearly lipid accumulation in the NC group. In contrast, cytoplasmic vacuoles were found in the liver sections of mice in the MC group, indicating that LBP partly mitigated the damage caused by diabetes. The hepatocytes structure of mice in the HG group was almost completely restored to normal. As shown in Figure 5E, in comparison with the NC group, the pathological damages of skeletal muscle sections in the MC group were partially ameliorated by LBP.

Figures 5D,F show the levels of glycogen in the liver and skeletal muscle, respectively. Glycogen levels in the MC group were significantly lower than that in the NC group (*p* < 0.05). LBP remarkably improved the contents of glycogen as compared with the MC group (*p* < 0.05), indicating that LBP promoted the accumulation of glycogen in diabetic mice. These findings disclosed that LBP effectively protected the liver, pancreas, and skeletal muscle from oxidative damage in diabetic mice.

### Effects of *Lycium barbarum* Polysaccharide on Gut Microbiota in Mice

#### Effects of *Lycium barbarum* Polysaccharide on Diversities of Gut Microbiota

**FIGURE 6:**
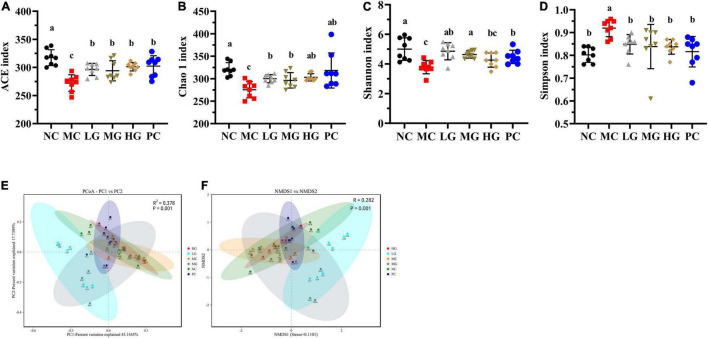
Effects of LBP on alpha diversity and beta diversity. **(A)** ACE index; **(B)** Chao 1 index; **(C)** Shannon index; **(D)** Simpson index; **(E)** PCoA; and **(F)** NMDS. Different letters denote significant differences at *p* < 0.05.

In Figures 6A,B, Chao 1 and ACE in the NC group were remarkably higher than those in the MC group (*p* < 0.05), illustrating that gut microbiota richness was significantly decreased in diabetic mice. LBP notably increased ACE and Chao 1 indexes in the LG, MG, and HG groups compared with the MC group (*p* < 0.05), indicating that LBP was effective in regulating the community richness of intestinal flora. Figures 6C,D show that Shannon was higher in the NC group than that in the MC group (*p* < 0.05), while the Simpson index was notably lower in the NC group (*p* < 0.05). We used principal coordinates analysis (PCoA) and non-metric multidimensional scaling (NMDS) to calculate beta diversity in this study. Figures 6E,F shows the results of PCoA in the MC group were different from those in the LG group. In the same way, NMDS analysis also showed a significant difference between the MC group and the LG group.

#### Effects of *Lycium barbarum* Polysaccharide on the Composition of Gut Microbiota

**FIGURE 7:**
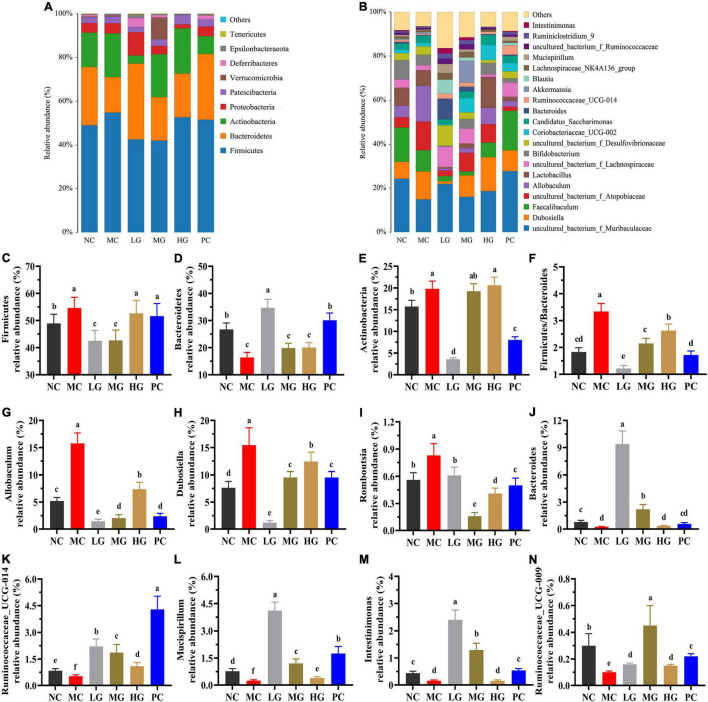
Effects of LBP on the relative abundance of gut microbiota at levels of phylum and genus. **(A)** Phylum (top 10); **(B)** genus (top 30); **(C)** Firmicutes; **(D)** Bacteroidetes; **(E)** Actinobacteria; **(F)** ratio of Firmicutes/Bacteroidetes; **(G)** Allobaculum; **(H)** Dubosiella; **(I)** Romboutsia; **(J)** Bacteroides; **(K)** Ruminococcaceae_UCG-014; **(L)** Mucispirillum; **(M)** Intestinimonas; and **(N)** Ruminococcaceae_UCG-009. Different letters denote significant differences at *p* < 0.05.

The top 10 phyla of intestinal flora are shown in Figure 7. In each group, the relative abundance of four predominant phyla was Firmicutes, Bacteroides, Actinobacteria, and Proteobacteria (Figure 7A), accounting for 95.77, 95.65, 91.59, 85.65, 95.16, and 94.18%, respectively. In Figure 7C, the relative abundance of Firmicutes was dramatically higher than that in the MC group compared to the NC, LG, and MG groups (*p* < 0.05). In Figure 7F, the ratio of Firmicutes–Bacteroides (F/B) in the MC group was higher than that in the NC group (*p* < 0.05). In this study, the top 30 genera were isolated in Figure 7B and analyzed in Figures 7G–N. As compared with the MC group, the relative abundance of *Allobaculum*, *Dubosiella*, and *Romboutsia* in the NC, LG, MG, HG, and PC groups were significantly decreased (*p* < 0.05), oppositely, the relative abundance of *Bacteroides*, *Ruminococcaceae_UCG-014*, *Mucispirillum*, *Intestinimonas*, *Ruminococcaceae_UCG-009* were remarkably increased (*p* < 0.05). These results indicated that LBP had a significant impact on the composition and structure of intestinal flora.

**FIGURE 8:**
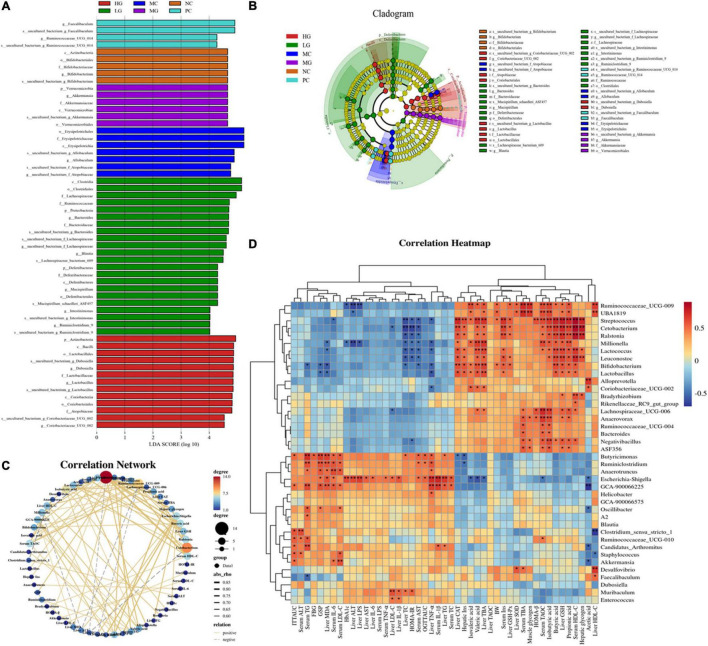
Comparison of gut microbiota by LEfSe analysis and correlation analysis. **(A)** Histogram of LDA values (lg 10 > 4); **(B)** taxonomic cladogram obtained by LEfSe. Differences are represented by the color of the most abundant class. **(C)** Visual network diagram. **(D)** Spearman correlation analysis between intestinal flora and biochemical profiles. Different letters denote significant differences at *p* < 0.05.

The linear discriminant analysis effect size (LEfSe) method was conducted to further identify the specific bacteria taxa (Figures 8A,B), and the calculation of effect size for variation-rich features was founded on linear discriminant analysis (LDA). In the NC, MC, LG, MG, HG, and PC groups (Figure 8A), we found 57 OTUs that were significantly different from other groups with 4, 7, 22, 6, 13, and 4 OTUs in each group, respectively. In the NC group, Actinobacteria (phylum level), Actinobacteria (class level), Bifidobacteriales (order level), Bifidobacteriaceae (family level), and *Bifidobacterium* (genus level) were more abundant, whereas Tenericutes (phylum level), Erysipelotrichia (class level), Erysipelotrichales (order level), Erysipelotrichaceae (family level), and *Allobaculum* (genus level) were enriched in the MC group. Proteobacteria and Deferribacteres (phylum level), Clostridia and Deferribacteres (class level), Clostridiales and Deferribacterales (order level), Lachnospiraceae, Ruminococcaceae, Bacteroidaceae, and Deferribacteraceae (family level), and *Bacteroides*, *Lachnospiraceae*, *Blautia*, *Mucispirillum*, *Intestinimonas*, and *Ruminclostridium_9* (genus level), *Lachnospiraceae_bacterium_09*, and *Mucispirillum_achaedleri_ASF457* (species level) were biomarkers in the LG group. Verrucomicrobia (phylum level), Verrucomicrobiae (class level), Verrucomicrobiales (order level), Akkermansiaceae (family level), and *Akkermansia* (genus level) were more abundant in the MG group. Actinobacteria (phylum level), Bacilli and Coriobacteriia (class level), Lactobacillales and Coribacteriales (order level), Lactobacillaceae and Atopobiaceae (family level), and *Dubosiella*, *Lactobacillus*, and *Coriobacteriaceae_UCG_002* (genus level) were enriched in the HG group. In the PC group, *Faecalibaculum* and *Ruminococcaceae_UCG_014* were enriched. These results indicated that LBP modulated the composition of intestinal flora.

### Effects of *Lycium barbarum* Polysaccharide on Short-Chain Fatty Acids in Mice Feces

In Table 3, the SCFAs levels in the MC group were significantly lower than that in the NC group (*p* < 0.05). As compared with the MC group, contents of acetate, propionate, butyrate, iso-butyrate, and total SCFAs were remarkably increased in the MG and HG groups (*p* < 0.05). Additionally, levels of valerate and iso-valerate in the HG group, as well as isovalerate in the MG group were increased as compared with the MC group (*p* < 0.05). These results illustrated that LBP significantly improved the levels of SCFAs in diabetic mice, particularly the contents of acetate, propionate, and butyrate.

**TABLE 3:** Effects of LBP on levels of SCFAs in mice feces.

	Group					
	
Item	NC	MC	LG	MG	HG	PC
Acetate (μmol/g)	10.52 ± 0.50 a	8.00 ± 0.28 c	7.59 ± 0.25 c	9.07 ± 0.55 b	10.76 ± 1.19 a	10.59 ± 1.05 a
Propionate (μmol/g)	9.49 ± 0.42 b	8.79 ± 0.18 c	9.88 ± 0.62 b	10.03 ± 0.72 b	10.86 ± 0.78 a	9.26 ± 0.29 c
Isobutyrate (μmol/g)	2.58 ± 0.14 a	2.28 ± 0.04 b	2.63 ± 0.23 a	2.74 ± 0.36 a	2.67 ± 0.10 a	2.50 ± 0.07 ab
Butyrate (μmol/g)	1.44 ± 0.07 b	1.26 ± 0.03 c	10.37 ± 0.02 bc	1.60 ± 0.07 a	1.65 ± 0.12 a	1.31 ± 0.07 c
Isovalerate (μmol/g)	1.24 ± 0.08 a	1.08 ± 0.02 b	1.14 ± 0.02 b	1.21 ± 0.06 ab	1.23 ± 0.07 a	1.17 ± 0.04 ab
Valerate (μmol/g)	1.21 ± 0.06 ab	1.02 ± 0.02 c	1.13 ± 0.03 b	1.15 ± 0.05 b	1.23 ± 0.09 a	1.10 ± 0.02 bc
Total SCFAs (μmol/g)	27.14 ± 1.95 a	22.94 ± 0.52 c	23.76 ± 1.65 b	24.78 ± 1.79 b	28.86 ± 1.45 a	27.51 ± 1.13 a

*Values are presented as the mean ± SD. Different letters denote significant differences at p < 0.05.*

### Potential Interactions Between Gut Microbiota and Biochemical Parameters

To assess the potential interaction between the gut microbiota and biochemical parameters, a Spearman’s correlation analysis was performed on the relative abundance of significantly changed gut microorganisms (Figure 8D, *p* < 0.05). Spearman’s rank analysis revealed that the relative abundance of *Ruminoccaceae_UCG-009*, *UBA1819*, *Streptococcus*, *Cetobacterium*, *Ralstonia*, *Millionella*, *Lactococcus*, *Leuconostoc*, *Bifidobacterium*, *Lactobacillus*, *Alloprevotella*, *Coriobacteriaceae_UCG-002*, *Bradyrhizobium*, *Rikenellaceae_RC9_gut_group*, *Lachnospiraceae_UCG-006*, *Anaerovorax*, *Ruminococcaceae_UCG-004*, *Bacteroides*, *Negativibacillus*, *ASF356* was negatively correlated with ITTAUC, OGTTAUC, FBG, GSP, HbA1c, HOMA-IR, as well as levels of TC, TG, LDL-C, ALT, AST, LPS, IL-6, IL-1β, TNF-α (serum and liver), while positively correlated with HOMA-β, BW, acetate, isovalerate, valerate, iso-butyrate, muscle glycogen, butyrate, propionate, and levels of Insulin, TBA, TAOC, HDL-C (serum and liver). Apart from that, the visual network of correlation analysis (Figure 8C) showed a close correlation between intestinal flora and biochemical profiles (| *r*| > 0.7). The results of correlation analysis showed that the relative abundance of *Cetobacterium*, *Millionella*, *Clostridium_sensu_stricto_1*, *Streptococcus*, *Ruminococcaceae_UCG_009* was negatively correlated with HOMA-IR and HDL-C, ALT, AST, TC, LPS in the liver. A significant positive correlation was observed between insulin (serum and liver) and *Cetobacterium*, *Streptococcus*, as well as *Ralstonia*. *Cetobacterium*, *Ruminiclostridium*, *Bifidobacterium*, and *Streptococcus* exhibited a positive correlation with HDL-C and serum insulin, liver glycogen, GSH, and CAT, as well as propionate, butyrate, and iso-butyrate. *Butyricimonas* was positively correlated with TG, GSP, and MDA. Similarly, *Ruminococcaceae_UCG-009* was positively correlated with TBA and TAOC (serum), HDL-C in the liver, muscle glycogen, iso-butyrate, and iso-valerate. Additionally, *Millionella* was negatively correlated with valerate, propionate, and TAOC (serum).

### Effects of *Lycium barbarum* Polysaccharide on the mRNA Expression of Colon and Liver in Mice

**FIGURE 9:**
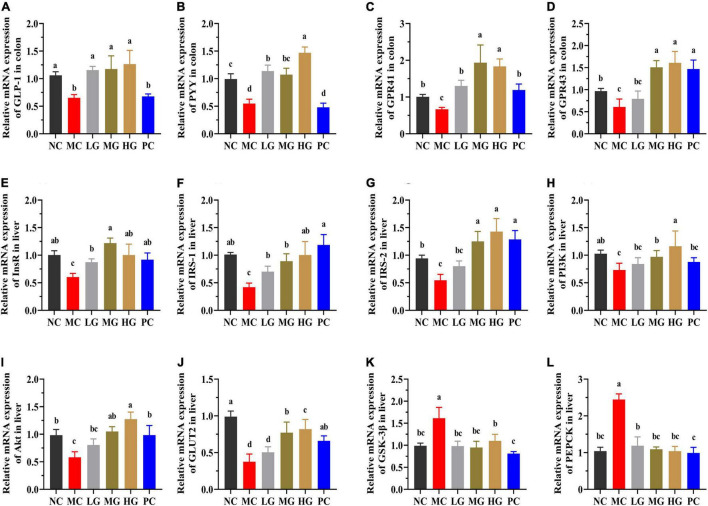
The mRNA expressions were determined using the real-time PCR and normalized with the β-actin expression. **(A)** GLP-1; **(B)** PYY; **(C)** GPR41; **(D)** GPR43; **(E)** InsR; **(F)** IRS-1; **(G)** IRS-2; **(H)** PI3K; **(I)** Akt; **(J)** GLUT2; **(K)** GSK-3β; and **(L)** PEPCK. Different letters denote significant differences at *p* < 0.05.

The underlying molecular mechanisms of LBP in diabetes alleviation were investigated using RT-PCR in this study. In Figures 9A–D, PYY, GLP-1, GPR43 (also known as free fatty acid receptor 2, FFAR2), and GPR41 (also known as FFAR3) relative expression levels were significantly higher in the NC, LG, MG, and HG groups as compared with those in the MC group (*p* < 0.05). In the MC group, the relative expression levels of insulin receptor (InsR), insulin receptor substrate-1 (IRS-1), insulin receptor substrate-2 (IRS-2), phosphatidylinositol 3-kinase (PI3K), protein kinase B (PKB, also known as Akt), and translocating glucose transporter-2 (GLUT2) were significantly lower than those in the NC group (*p* < 0.05). LBP reduced the levels of InsR (Figure 9E), IRS-1 (Figure 9F), IRS-2 (Figure 9G), PI3K (Figure 9H), Akt (Figure 9I), and GLUT2 (Figure 9J) in the LG, MG, and HG groups compared with the MC group (*p* < 0.05). In contrast, the MC group had a significantly higher relative expression of phosphorylated glycogen synthase kinase-3β (GSK-3β) (Figure 9K) and phosphoenolpyruvate carboxykinase (PEPCK) (Figure 9L) than those in the NC, LG, MG, and HG groups, respectively (*p* < 0.05). These results indicated that LBP effectively regulated the expression levels of genes involved in blood glucose modulation in diabetic mice.

## Discussion

In this study, we assessed the LBP’s hypoglycemic and hypolipidemic activity, as well as its effects on the gut microbiota of diabetic mice. The yield of LBP in this study is 2.76 ± 0.33%, which is slightly lower than that in previously reported study (3.08%) (25). Carbohydrates (62.27 ± 2.37%), uronic acid (25.03 ± 1.27%), and protein (2.92 ± 0.17%) predominate in LBP, which is consistent with previous research with 60.96 ± 5.58, 20.98 ± 2.49, and 5.30 ± 0.12%, respectively (20). According to a previous report (19), the spectroscopy results disclosed that LBP was a complex glycoconjugate composed of acidic heteropolysaccharides and polypeptides. LBP is primarily composed of Rha, Ara, Xyl, Man, Glc, Gal, GlcA, and GalA residues with a molar ratio of 0.23: 1.90: 0.26: 0.20: 1.0: 1.26: 0.44: 1.49. And Ara, Gal, and GlcA are dominant in it, these results are consistent with the reported research on LBP (20). Polysaccharide physicochemical properties are closely linked to their MW, and only polysaccharide fractions in the appropriate MW mass range of the same source have the best biological activities (44). According to a published review, LBP had a MW range of 10–2300 kDa (24). Furthermore, a previous study reported the identification of numerous pharmacologically active polysaccharides with MWs ranging from 3.5 to 160 kDa (45). In this study, the MW of LBP is 98.0 kDa, which is closed to the specified range, indicating that LBP may have an excellent biological function. The hypoglycemic results of LBP in this study have confirmed the conclusion.

In the animal experiments, we conducted OGTT and ITT (Supplementary Figure S1) in HFD-induced mice to ensure that the diabetic mice employed in this study exhibited significant insulin resistance and typical T2DM characteristics before LBP intervention. In diabetic mice, LBP treatment significantly reversed the typical symptoms, including the decrease of FBG, decrease in food consumption and water intake, as well as prevention of BW loss. When compared with the NC group, these profiles in the MC group were significantly higher. A previous study found that weight loss in diabetics was caused by impaired energy metabolism, blood glucose imbalances, and underutilization of carbohydrates (46). In diabetic mice, LBP effectively regulated the levels of BW, FBG, as well as food and water intake. These findings demonstrated that LBP elevated energy metabolism by increasing the uptake and utilization of blood glucose in diabetic mice. LBP not only markedly restored the impaired glucose tolerance but also improved the insulin tolerance in diabetic mice. The levels of HbA1c and GSP reflected the average blood glucose level over 2–3 weeks (47). In comparison with the MC group, LBP reduced HbA1c and GSP levels in the LG, MG, and HG groups, implying that LBP continuously maintained blood glucose homeostasis under control. Historically, hyperglycemia has been linked to hyperinsulinemia, the possible reason is that the host requires more insulin to maintain glucose homeostasis (7). As a result, the maintenance of insulin concentrations is critical for maintaining blood glucose homeostasis (48). LBP significantly improved the levels of insulin secretion and sensitivity, as well as alleviated insulin resistance in diabetic mice. The liver and skeletal muscle are the central target organs of insulin and play a vital role in the maintenance of glucose homeostasis (49). LBP promoted glycogen accumulation in diabetic mice, both in the liver and skeletal muscle, and the probable reason is that insulin facilitates glycogen synthesis or inhibits hepatic gluconeogenesis (7). Consequently, the pancreas, liver, and skeletal muscles are indispensable tissues or organs for mitigating diabetes (50). In this study, H&E staining revealed that LBP restored the pathological changes in the pancreas, liver, and skeletal muscle caused by diabetes.

Type 2 diabetes mellitus is frequently associated with complications, which pose a greater threat to diabetics than diabetes itself (51). As a result, reducing diabetes-related complications is critical to improving diabetes (4). According to a reported study, high levels of TC, TG, and LDL-C may cause intestinal endothelial dysfunction and worsen the onset and progression of atherosclerosis (52). TBA, ALT, and AST levels are regarded as critical indicators of hepatocellular injury and liver dysfunction (53). LBP increased the levels of TBA and HDL-C while decreasing TC, TG, LDL-C, ALT, and AST levels. The reported literature has proved that oxidative stress can cause cardiovascular disease, diabetic nephropathy, and insulin dysfunction in the host, increasing the risk of developing diabetes (54). LBP increased the activities of CAT, SOD, and GSH-Px, while decreasing MDA levels, indicating that LBP acted as a hepatoprotective agent and mitigated oxidative damage in diabetic mice. Furthermore, LBP reduced inflammatory factors, such as IL-1β, IL-6, and TNF-α (serum and liver), which is consistent with previous studies of *G. lucidum* polysaccharide in alleviating diabetic mouse inflammation (16, 17). LPS is primarily derived from the metabolism of certain intestinal microorganisms and can cause inflammation and insulin resistance in target tissues and organs (55). In addition to this, the host’s chronic hyperglycemia and hyperlipidemia may result in the production of excessive ROS (56).

The gut microbiota is important in the treatment of T2DM (57). The biodiversity of gut microbiota in the MC group of mice was significantly lower than that in the NC group. A previous study reported that the increased ratio of the phyla Firmicutes–Bacteroidetes was associated with obesity and the balance of intestinal flora (58). The F/B ratio is critical in reflecting the impact of gut flora on health, and an elevated ratio frequently indicates an unhealthy physiological state *in vivo* (59). In this study, LBP significantly decreased F/B levels in the LG, MG, and HG groups when compared with the MC group (*p* < 0.05), indicating that the LBP’s hypoglycemic effects may be mediated by regulating the composition of intestinal microbiota in diabetic mice. By comparing specific bacteria, we investigated the modulatory effect of LBP on the microbiome. In comparison with the MC group, the relative abundance of *Bacteroides*, *Ruminococcaceae_UCG-014*, *Ruminococcaceae_UCG-009*, *Mucispirillum*, and *Intestinimonas* was significantly higher in the NC group, while *Allobaculum*, *Dubosiella*, and *Romboutsia* were significantly lower (*p* < 0.05). LBP reversed the decrease of *Bacteroides*, *Ruminococcaceae_UCG-014*, *Mucispirillum*, *Intestinimonas*, *Ruminococcaceae_UCG-009*, and the increase of *Allobaculum*, *Dubosiella*, *Romboutsia* in the LG, MG, and HG groups as compared with the MC group (*p* < 0.05). *Bacteroidetes* is a vital Bacteroidetes phylum member that increased in LBP-treated mice compared with the MC group. A previous study found that *Bacteroides* improved metabolic function and immune dysfunction in HFD-induced mice (60). The increased relative abundance of *Bacteroides* is consistent with a previously reported study (61). *Ruminococaceae* degraded and fermented LBP, which was then used for its growth and predominance. Additionally, certain *Ruminococaceae* genera can produce both butyrate and acetate, which is advantageous for *Roseburia* to produce butyrate (62). LBP contains many glycosides and has been shown to restrain the decline of *Ruminococaceae_UCG-014* and *Ruminococcaceae_UCG-009* in diabetic mice (Figures 7K,N). In this study, *Ruminococaceae_UCG-014* is a SCFAs-producing bacteria that was enriched by LBP (62). *Mucispirillum*, which lives in the gut mucous layer of rodents’ guts, uses SCFAs for energy metabolism but doesn’t produce them (62). A recent study found that *Mucispirillum schaedleri* protected mice from colitis by antagonizing *Salmonella* (63). LBP increased *Mucispirillum* abundance in this study (Figure 7L). *Intestinimonas*, a butyrate-producing genus found in the mouse intestine (64), was enriched in LBP-treated groups (Figure 7M). *Allobaculum* is an important functional phylotype of metabolic dysbiosis, as well as a harmful genus of bacteria that is more prevalent in diabetics (65). The relative abundance of *Allobaculum* remarkably increased in the MC group but decreased in LBP-treated groups as compared with that in the NC group, indicating that LBP effectively decreased the relative abundance of *Allobaculum* in diabetic mice. A previous study reported that *Grifola frondosa* heteropolysaccharide increased the relative abundance of *Allobaculum*, implying that it may helped the host to fight against non-alcoholic fatty liver disease (NAFLD) (66). On the contrary, the proportion of *Allobaculum* in LBP-treated groups was significantly lower than that in the MC group (*p* < 0.05). *Romboutsia* is another SCFAs-producing genus that is regarded to have beneficial effects on the regulation of intestinal homeostasis (67, 68). It has also been linked to circulating inflammatory (IL-1β) and behavioral outcomes, such as lethargy, and anxiety-like behavioral (67). In this study, *Sargassum fusiforme* polysaccharide caused an increase in *Romboutsia* (68), whereas LBP decreased it in this study. These disparate or opposite results of *Allobaculum* and *Romboutsia* suggested that the same genus could play different roles under different physiological states. A reported study showed that *Dubosiella* was positively related to acetate (69). LBP inhibited the increase of *Dubosiella* in diabetic mice in this study, which was consistent with another study (70). To summarize, LBP improved the abundance of beneficial bacteria, while decreasing the abundance of harmful bacteria, protecting the host from damages caused by harmful microorganisms and their metabolites. Changes in the composition of intestinal flora have been linked to the emergence and progression of diabetes (71). Recent studies disclosed that many metabolic diseases were caused by the changes in gut microbiota, and reshaping intestinal flora was critically important to the host’s health (72). As a result, we investigated the relationship between intestinal flora and biochemical profiles. The relative abundance of *Cetobacterium*, *Millionella*, *Clostridium_sensu_stricto_1*, *Streptococcus*, *Ruminococcaceae_UCG-009* was negatively correlated with HDL-C, HOMA-IR, ALT, AST, TC, and LPS in the liver. A positive and significant correlation was found between insulin (serum and liver) and *Cetobacterium*, *Streptococcus*, as well as *Ralstonia*. *Cetobacterium*, *Ruminiclostridium*, *Bifidobacterium*, and *Streptococcus* showed a positive correlation with HDL-C and insulin (serum), hepatic glycogen, GSH, and CAT in the liver. Furthermore, *Bifidobacterium* was involved in liver injury and inflammation. These results indicated that *Cetobacterium*, *Millionella*, *Clostridium_sensu_stricto_1*, *Streptococcus*, *Ruminococcaceae_UCG-009*, *Cetobacterium*, *Streptococcus*, *Ralstonia*. *Cetobacterium*, *Ruminiclostridium*, *Bifidobacterium*, and *Streptococcus* may be related to hepatic injury and insulin resistance. Our study demonstrated that LBP not only alleviated inflammation, and altered the composition of intestinal flora, but also improved the production of SCFAs. SCFAs are important metabolites that are produced by beneficial microorganisms during the fermentation of indigestible polysaccharides and fibrates (73, 74). SCFAs influence glucose homeostasis by modulating glucose absorption and utilization in metabolically active tissues or organs (75). Acetate, propionate, and butyrate are metabolic substrates for the synthesis of cholesterol, and gluconeogenesis is important for controlling blood glucose, cholesterol, and lipid metabolism (76). Furthermore, propionate inhibits cholesterol and fatty acid synthesis in the liver (14). Butyrate is the most abundant energy substance in colonocytes and has a variety several beneficial effects, including the activities of immunomodulation, anti-inflammatory, and antimicrobial properties (77). The rise of acetate, propionate, and butyrate corresponded to the effects of LBP on the alleviation of hypertriglyceridemia, hyperglycemia, and hypercholesterolemia in T2DM mice. Isobutyric acid and isovaleric acid are byproducts of protein fermentation, most notably microbial protein fermentation (78). The possible cause for the increase of the isobutyric acid and isovaleric acid could be related to the content of protein in LBP (79). In this study, SCFAs changes in diabetic mice were linked to LBP modulation in *Bacteroides*, *Ruminococcaceae_UCG-014*, *Intestinimonas*, *Ruminococcaceae_UCG-009*, and *Romboutsia*. In addition to this, the genera *Allobaculum*, *Mucispirillum*, and *Dubosiella* could not be classified as either pro-inflammatory or anti-inflammatory bacteria (80). These results suggested that reshaping the composition and structure of the gut microbiota was an important strategy for diabetes risk reduction.

Several studies have shown that SCFAs helped to improve energy metabolism. SCFAs can directly activate G-protein-coupled receptors (GPCRs), mainly including GPR109A, GPR43, and GPR41 (8). Both GPR41 and GPR43 could be activated by SCFAs, particularly acetate and propionate (8). After stimulation by SCFAs, GPR41, and GPR43 could enhance the secretion of GLP-1 and PYY from intestinal L cells (8). The increased GLP-1 and PYY expression may improve insulin sensitivity and inhibits gluconeogenesis by promoting insulin secretion (37, 81). In this study, LBP significantly increased the expression of GPR41, GPR43, GLP-1, and PYY, while decreasing HOMA-IR. Insulin resistance of the liver has been linked to the onset and progression of T2DM, and it is widely acknowledged that the regulation of some critical metabolic pathways may be an excellent treatment option (68). The IRS/PI3K/Akt signaling pathway is widely acknowledged to play a critical role in the transmission of insulin signals and modulation of glucose metabolism (7, 68). IRS-1 and IRS-2 are downstream signal factors of InsR, and those are the critical kinase of glucose uptake and the target of insulin resistance (13). The increased insulin could stimulate the phosphorylation of InsR, promoting IRS-2 binding to PI3K while inhibiting IRS-1 signal transmission (13). The dysfunction of PI3K may affect the process of activated Akt, causing disruptions in glucose metabolism (68). In addition to this, insulin stimulation activates phosphorylated Akt, which not only improves glucose uptake by promoting GLUT2 to the hepatocyte membrane but also regulates the inhibition of GSK-3β in regulating the process of glycogen synthesis (13, 68). In this study, LBP significantly increased the expression of InsR, IRS-1, IRS-2, PI3K, and Akt in the liver of diabetic mice. LBP increased blood glucose absorption and utilization in diabetic mice, indicating that diabetes may be alleviated by enhancing IRS/PI3K/Akt signaling pathways. Apart from this, PEPCK is a gluconeogenic enzyme and is elevated in diabetics (82). In addition, hyperglycemia is caused by excessive hepatic glycogen and glucose production. LBP decreased the relative expression of PEPCK in this study. Particularly, the expression of IRS-1, IRS-2, PI3K, GLUT2, and Akt was significantly higher in the HG group than those in the LG and MG groups, demonstrating that the HG group’s superior ability to modulate the glucose production of high-dosage LBP. As a result, LBP reduced insulin resistance in T2DM mice by promoting the absorption and utilization of excess blood glucose and suppressing gluconeogenesis.

## Conclusion

In this study, LBP significantly reduced food and water intake, alleviated hyperglycemia and insulin resistance, decreased lipid accumulation, and inhibited weight loss in diabetic mice by increasing blood glucose uptake and utilization. LBP increased the activities of SOD, CAT, and GSH-Px, indicating that LBP could protect the liver and pancreas from oxidative damage in diabetic mice. The 16S rDNA analysis illustrated that LBP significantly changed the intestinal composition, decreasing *Allobaculum*, *Dubosiella*, and *Romboutsia*, increasing *Bacteroides*, *Ruminococcaceae_UCG-014*, *Mucispirillum*, *Intestinimonas*, and *Ruminococcaceae_UCG-009*. The SCFAs level in LBP-treated mice was significantly increased, which was accompanied by the increase in SCFAs-producing genera. The RT-PCR analysis revealed that LBP up-regulated the expressions of GLP-1, PYY, GPR41, GPR43, activated InsR/PI3K/AKT signal pathway, and down-regulated the expressions of GSK-3β and PEPCK of diabetic mice. In conclusion, these findings suggested that LBP may alleviate hyperglycemia and hyperlipidemia by modulating intestinal flora and could be used as a beneficial ingredient adjuvant in T2DM.

## Data Availability Statement

The raw sequencing data uploaded at https://www.ncbi.nlm.nih.gov/Traces/study/?acc=PRJNA826571.

## Ethics Statement

The animal study was reviewed and approved by the Animal Care Committee of Xi’an Jiaotong University.

## Author Contributions

QM contributed to conceptualization, wrote the original draft, and investigation. RZ, TC, and ZZ investigated the study. XX contributed to data curation. HL contributed to software. CN, XY, AT, BT, and ZC contributed to software and validation. MZ validated the study. JL contributed to methodology and revising and supervising the study. All authors contributed to the article and approved the submitted version.

## Conflict of Interest

The authors declare that the research was conducted in the absence of any commercial or financial relationships that could be construed as a potential conflict of interest.

## Publisher’s Note

All claims expressed in this article are solely those of the authors and do not necessarily represent those of their affiliated organizations, or those of the publisher, the editors and the reviewers. Any product that may be evaluated in this article, or claim that may be made by its manufacturer, is not guaranteed or endorsed by the publisher.
