# Supplementary material for: Hypoglycemic Effects of Lycium barbarum Polysaccharide in Type 2 Diabetes Mellitus Mice via Modulating Gut Microbiota
Source: Front Nutr. 2022 Jun 30;9:916271. doi: 10.3389/fnut.2022.916271 (PMC9280299; doi:10.3389/fnut.2022.916271)
Supplement: Supplementary file 1 [file Data_Sheet_1.docx]

Supplementary materials

Fig. S1. Effects of HFD on OGTT, OGTTAUC, ITT, and ITTAUC.

(a) OGTT; (b) OGTTAUC; (c) ITT; (d) ITTAUC. Values are presented as the mean ± SD.

Different letters denote significant differences at *p* < 0.05.

Table S1 Sequences of primers used for RT-PCR in this study

|  | Primer sequence | |
| --- | --- | --- |
| Gene name | Forward primer (5’-3’) | Reverse primer (5’-3’) |
| GLP-1 | TTACTTTGTGGCTGGATTGCTT | AGTGGCGTTTGTCTTCATTCA |
| PYY | ACGGTCGCAATGCTGCTAAT | GACATCTCTTTTTCCATACCGCT |
| GPR41 | TTGCTAAACCTGACCATTTCGG | GATAGGCCACGCTCAGAAAAC |
| GPR43 | TGCTACGAGAACTTCACCCAA | CACACGAAGCGCCAATAACAG |
| InsR | CAATGGTGCTGAGGACACTAGG | GTGCTCTTCGTGGCTTGTGG |
| IRS-1 | CGATGGCTTCTCAGACGTG | CAGCCCGCTTGTTGATGTTG |
| IRS-2 | CTGCGTCCTCTCCCAAAGTG | GGGGTCATGGGCATGTAGC |
| PI3K | CATAACCTGCAAACACTGCCC | ATCCTGCAAGGACATATTGTTGT |
| Akt | ATGAACGACGTAGCCATTGTG | TTGTAGCCAATAAAGGTGCCAT |
| GLUT2 | CTCTGTGCTGCTTGTGGAGA | CGGCACAGAAAAACATGCCA |
| GSK-3β | TCGTCCATCGATGTGTGGTC | TTGTCCAGGGG TGAGCTTTG |
| PEPCK | CTGCATAACGGTCTGGACTTC | CAGCAACTGCCCGTACTCC |
| β-actin | GGCTGTATTCCCCTCCATCG | CCAGTTGGTAACAATGCCATGT |

Table S2 Effects of LBP on food intake, water intake, body weight, and FBG in mice

|  | Group | | | | | |
| --- | --- | --- | --- | --- | --- | --- |
| Item | NC | MC | LG | MG | HG | PC |
| Initial BW. (g/mice) | 25.10±0.92 b | 29.20±2.19 a | 29.18±1.90 a | 29.21±1.87 a | 29.32±1.95 a | 29.05±1.97 a |
| Terminal BW. (g/mice) | 29.07±1.92 a | 24.58±1.03 b | 25.36±1.32 b | 25.78±1.89 b | 26.21±1.60 b | 26.73±1.67 b |
| Initial FBG (mmol/L) | 6.88±0.40 b | 15.63±1.45 a | 15.18±1.27 a | 15.48±1.63 a | 15.94±1.49 a | 15.28±1.50 a |
| Terminal FBG (mmol/L) | 6.94±0.67 c | 17.32±1.62 a | 13.12±1.25 b | 12.90±1.02 b | 11.60±1.10 b | 12.73±1.36 b |
| Total food Intake (g/mice) | 32.10±1.33 c | 39.47±1.93 a | 37.48±1.42 ab | 35.86±1.81 b | 33.90±1.30 bc | 34.82±1.89 bc |
| Total water intake (mL/mice) | 56.86±4.48 c | 77.40±4.38 a | 64.45±3.04 b | 61.80±3.50 bc | 60.27±3.63 bc | 67.30±4.79 b |

Values are presented as the mean ± SD. Different letters denote significant differences at *p* < 0.05.
